# Clinical Potentials of miR-576-3p, miR-613, NDRG2 and YKL40 in Colorectal Cancer Patients

**DOI:** 10.31557/APJCP.2020.21.6.1689

**Published:** 2020-06

**Authors:** Manal Nasreddin Eldaly, Fateheya Mohamed Metwally, Wafaa Ghoneim Shousha, Abeer Salah EL-Saiid, Shimaa Shawki Ramadan

**Affiliations:** 1 *Department of Biochemistry, Faculty of Science, Helwan University, Cairo, Egypt. *; 2 *Department of Environmental & Occupational Medicine, National Research Centre, Cairo, Egypt. *; 3 *Department of Clinical & Chemical Pathology, National Cancer Institute, Cairo University, Cairo, Egypt. *

**Keywords:** micro RNA, colon cancer, diagnostic, molecular biomarkers

## Abstract

**Introduction:**

Colorectal cancer (CRC) is the most common type of gastrointestinal tract cancers. This investigation aim was to assess the expression of* miR-576-3p* and *miR-613* in CRC patients in addition to NDRG2 and YKL40 serum levels determination to decide their diagnostic and prognostic significance.

**Methods:**

Sixty early diagnosed CRC patients prior to any treatment in addition to twelve healthy subjects were enrolled in this study. Blood samples were taken from subjects and allowed for clotting and centrifugation, then the collected sera were stored at -80ºC till it were used for detection of our molecular biomarkers. The mature *miRNAs* expressions (*miR-576-3p *and *miR-613*) were detected in serum by qRT-PCR, while NDRG2 and YKL40 serum levels were determined by ELISA. In addition, the correlation of the measured parameters with the clinicopathological data of the patients was investigated.

**Results:**

The study results showed that both miRNA-576-3p and miRNA-613 were down-regulated in CRC patients with fold change 0.33, 0.36; respectively. A significant positive correlation was observed between miR-576-3p and miR-613 (r = 0.75, p < 0.001). NDRG2 serum levels were decreased in patients compared to the control group but the decrease wasn’t statistically significant. On the other hand, it was observed that YKL40 serum level was significantly increased in CRC patients compared to control (p-value < 0.001). Furthermore, YKL40 showed a very high diagnostic value (AUC = 0.97, specificity = 91.7%, sensitivity = 96%, p-value = 0.0001).

**Conclusion:**

The observations of this investigation concluded that, the expressions of miR-576-3p and miR-613 in addition to YKL40 serum levels determinations may help in the diagnosis of CRC.

## Introduction

Over one million new patients of colon cancer are diagnosed every year causing death for more than 800,000 (Bray et al., 2018). Early diagnosis can decrease the mortality rate of the CRC by 60% (He and Efron, 2011). Screening tests mainly relies upon colonoscopy and fecal occult blood testing (FOB) (Cunningham et al., 2010). Colonoscopy has many disadvantages such as high cost, invasiveness and radiation exposure. The FOB test has low sensitivity, so new non-invasive markers with high specificity and sensitivity are critically required for CRC screening. 

Micro RNA (miRNA) is a short single-stranded non-coding RNA (containing about 22 nucleotides) (Chen et al., 2012). It plays important roles in gene expression post-transcriptional regulation and RNA silencing. miRNAs are believed to be disease-specific and they were determined in serum by using quantitative real-time polymerase chain reaction (qPCR) technique (Lv et al., 2017). Altered expression of a specific miRNA may explain the effects of oncogene and tumor suppressor. There are several studies have been published which study the diagnostic roles of miRNAs in CRC (Hollis et al., 2015). In human, around 2000 variant miRNAs have been characterized according to miRBase (Kozomara and Griffiths-Jones 2013). miR-576-3p might be considered as a prognostic marker of breast cancer consequent to the chemotherapy resistance development (Lv et al., 2014). It was demonstrated that miR-576-3p expression was diminished in patients with leukemia (Coskun et al., 2013) and basal cell carcinoma (Al-Eryani et al., 2018). miR-576-3p was additionally founded to be in low expression in sera of patients with non-melanoma skin malignancy (Balci et al., 2016). miR-613 plays a role in the pathogenesis of several sorts of cancers as it had been found to down-regulate the invasion and proliferation of hepatocellular carcinoma (Wang et al., 2016). It has an anti-oncogene role in many types of cancers, such as breast cancer (Wu et al., 2019), prostate cancer (Ren et al., 2016), non-small cell lung cancer (Li et al., 2016), and papillary thyroid carcinoma (Qiu et al., 2016).

NDRG2 (N-myc downstream-regulated gene 2) is one of the members of NDRG family (NDRG1-4) and it is considered to be a tumor suppressor gene (Oh et al., 2012). Levels of *NDRG2* expression are decreased in CRC and high-risk adenoma (Lorentzen et al., 2007). It was stated that, patients with low levels of NDRG2 mRNA have diminished disease-free survival duration and decreased overall survival duration than cases with preserved *NDRG2 mRNA* expression (Chu et al., 2011).*NDRG2* expression was mainly determined in the liver, colon, muscle, heart and brain (Hu et al., 2006).YKL-40 (Chitinase 3-like 1) is a highly conserved glycoprotein created by cancer cells (involving CRC cells), neutrophils and macrophages (Johansen et al., 2009) and by embryonic stem cells (Johansen et al., 2007).YKL-40 acts as an anti-apoptotic marker, renovating of extracellular matrix, and inﬂammation (Faibish et al., 2011). So, serum YKL-40 can provide some aspects about tumor growth and spreading than the already used classic markers (Bojesen et al., 2011).

This study was designed to detect serum levels of miR-576-3p, miR-613, NDRG2 and YKL40 in colon cancer patients to estimate their diagnostic value which may help to understand their roles in the pathogenesis of CRC. In addition, the study was extended to investigate the correlation of parameters with the clinicopathological features of the patients.

## Materials and Methods

*Subjects*

The serum samples from sixty CRC patients were collected from the National Cancer Institute, Cairo University, Egypt during the period from July 2016 to January 2017. All samples were obtained from early diagnosed patients preceding any treatment. Twelve healthy sex and age-matched subjects were also enrolled in this examination and considered as the control group. Five ml of venous blood samples were gathered from patients and control then partitioned into 2 volumes after centrifugation to obtain serum: a part of serum for the examination of miRNAs expression and another for analysis of NDRG2 and YKL-40. All samples were stored at - 80ºC. The clinicopathological data of the patients were obtained to be correlated with miRNAs, NDRG2 andYKL40. Witten informed consents were obtained from subjects in this study. 

*Methods*

*RNA preparation and real-time PCR for determination of miR-576-3p and miR-613*

Total RNA was isolated from the frozen serum samples by utilizing QIAamp RNA serum Mini Kit -according to guidelines with Cat. No .52304 (Qiagen, Düsseldorf, Germany). Then cDNA synthesis was prepared from 100 ng of the extracted RNA, by utilizing a kit RevertAid Reverse Transcriptase (Thermo Fisher). Cat. No. EP0441 -as indicated by the producer’s guidelines. Then cDNAs were exposed to the qRT-PCR (quantitative reverse transcription polymerase chain reaction) utilizing Quantitect SYBR green PCR kit with Cat. No .204141- as indicated by the producer’s guidelines to detect miR-576-3p and miR-613 (Table 1).

Specific primers for miR-576-3p, miR-613 and housekeeping gene (Human GAPDH) are appeared in Table 2. The GAPDH gene was utilized as an endogenous control for miRNA expression. The relative expression levels of miR-576-3p and miR-613 were determined using the comparative delta Ct (2^−ΔΔCT^) manner.

Amplification curves and Ct values were determined by the strata gene MX3005P software. To assess the variation of expression on the micro RNA of the different samples, the CT of each example was contrasted with that of the control as indicated by the “ΔΔCt” method. 

*Determination of NDRG2 and YKL40 levels*

NDRG2 and YKL40 serum levels were assessed by quantitative sandwich enzyme-linked immunosorbent assay (Kono Biotech, Co., Ltd with Cat. No. KN2289Hu; RandD systems, Inc, biotechne brand, Cat No. DC3L10, respectively) in accordance with the producer’s directions. The reaction was ended by the introducing of stopping reagent and the change of color was determined spectrophotometrically at 450 nm for NDRG2 and 570 nm for YKL40. The concentrations of NDRG2 and YKL40 in the samples were estimated by comparing the optical density (OD) of the samples to the standard curve. 

*Statistical analysis*

SPSS© Statistics version 17 (IBM© Corp., Armonk, NY, USA) was used to statistically analyze the data of the study. Quantitative parameters were expressed as mean ±SEM, while qualitative data were expressed as percentage. For quantitative data, independent sample t-test was performed in the comparison between two groups. While one way ANOVA test was performed for the comparison between three groups. The correlations between numerical variables were estimated by Pearson’s correlation coefficient (r) determination. All p-values were two-sided. P-values < 0.05 were considered as statistically significant.

## Results

Patients age ranged from 29 year to 60 year (32 males and 28 females) by mean level 45.77 and the age mean of the healthy individuals (2 males and 10 females)was 40.67 (p-value = 0.170).The patients’ characteristics as they are classified according to grade into 86.96% grade II and 13.04% grade III. Their stages classified according to the TNM system as stage IIA was the highest percentage (30.43%). However the lowest percentage was 4.35% for IIB and IIIC. The classification according to tumor site was 65.22% in colon, 30.43% in rectum and 4.35% in colon and rectum together. Patients with positive lymph node metastasis represented 56.52% compared to those with negative lymph node metastasis43.48%.

It was observed that, miR-576-3p delta Ct was significantly increased in CRC patients (3.97) when compared to control (2.38) (p-value = 0.002). Also, miR-613 delta Ct for patients (4.15) was higher than that of the control (2.69) and the difference was statistically significant (p value=0.003) (Table 3). Data in Table 3 also showed that NDRG2 was decreased in patients compared to control but the decrease wasn’t reach to the significance level. On the other hand, it was observed that YKL40 was significantly increased in patients (3813.40) compared to control (731.50) (p-value < 0.001).It was illustrated that, the expression of miRNA-576-3p was down-regulated in patients compared to control (Fc = 0.33) (Table 4). Also, the expression of miR-613 was down-regulated in patients compared to control (Fc = 0.36) (Table 5). 

Data in Table 6 illustrated that, the expression of miR-576-3P was decreased in grade III compared to grade II but the decrease wasn’t statistically significant. The same was observed for miR-613 which was decreased in grade III compared to grade II. The data also clarified that, miR-576-3P expression in positive lymph node metastasis patients was lower than that of the negative lymph node metastasis patients but the decrease was regarded as statistically non-significant (p-value =0.12). The same was for miR-613 expression as it was decreased in positive lymph node metastasis patients compared to negative lymph node metastasis patients (p-value=0.14).

A significant negative correlation was observed between miR-613 and CA19-9 (r=-0.27, p=0.04). Also, a significant negative correlation between NDRG2 and CEA was observed (r= -0.37, p=0.004).On the other hand, there was a significant positive correlation between miR-576-3p and miR-613 (r=0.75, p<0.001)(Table 7).

**Figure 1:**
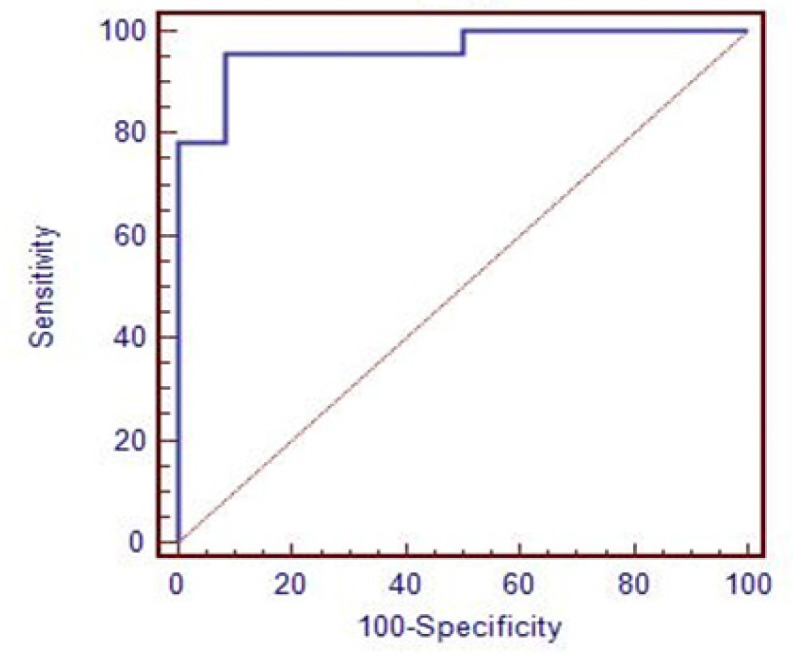
ROC Curve of YKL40 in CRC Patients Compared to Control; - Specificity: 91.7%, Sensitivity: 96%, Cut off: > 1200, AUC: 0.97

**Figure 2:**
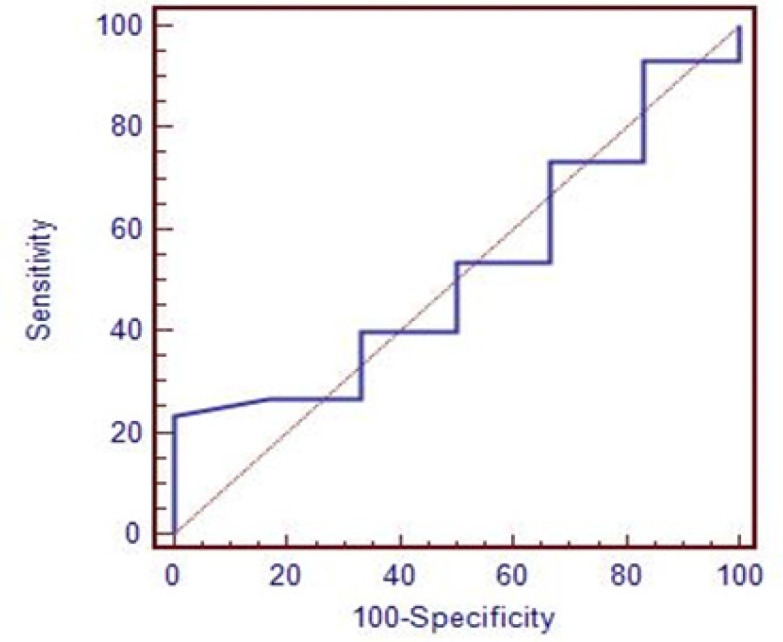
ROC Curve of NDRG2 in CRC Patients Compared to Control; -Specificity: 66.7%, Sensitivity: 60%, Cut off: > 2.5, AUC: 0.54

As illustrated in Table 8 and Figure 1, YKL40 showed a very high diagnostic value (AUC = 0.97, specificity=91.7%, sensitivity=96%, p-value = 0.0001) in opposite to NDRG2 which showed a very low diagnostic value (AUC =0.54) (Figure 2).

**Table 1:** Cycling Conditions for SYBR Green Real-Time PCR According to Quantitect SYBR Green PCR kit

Gene	Reverse transcription	Primary denaturation	Amplification (40 cycles)			Dissociation curve (1 cycle)		
			Secondary denaturation	Annealing (Optics on)	Extension	Secondary denaturation	Annealing	Final denaturation
*GAPDH*	50˚C	94˚C	94˚C	58˚C	72˚C	94˚C	58˚C	94˚C
	30 min.	5 min.	15 sec.	30 sec.	30 sec.	1 min.	1 min.	1 min.
*miR-613*	50˚C	94˚C	94˚C	60˚C	72˚C	94˚C	60˚C	94˚C
	30 min.	5 min.	15 sec.	30 sec.	30 sec.	1 min.	1 min.	1 min.
*miR-576-3P*	50˚C	94˚C	94˚C	60˚C	72˚C	94˚C	60˚C	94˚C
	30 min.	5 min.	15 sec.	30 sec.	30 sec.	1 min.	1 min.	1 min.

**Table 2:** RNA Specific Primers for miR-576-3p, miR-613 and Housekeeping Gene (Human GAPDH)

Gene	Primer sequence (5'-3')	Reference
*Human GAPDH*	CTCTGATTTGGTCGTATTGGG	(Li et al., 2014)
	TGGAAGATGGTGATGGGATT	
*miR-576-3P*	AAGATGTGGAAAAATTGGAATC	(Liang et al., 2015)
	ATTCTAATTTCTCCACGTCTTT	
*miR-613*	CCGCTCGAGTCTACTAGGTGTGGGCTTTA	(Ou et al., 2011)
	CCCAAGCTTCTGTGGCCTTCCTTACTCTT	

**Table 3:** The Mean Levels of Parameters of the Patients Compared with Those of Control

Parameters	Patients Mean ±SE	Control Mean ±SE	*P*-value
HB (gm/dl)	10.49 ± 0.20	13.32 ± 0.30	<0.001*
Platelets (x10^3^/mm^3^)	414.63 ± 20.52	291.50 ± 18.12	0.1
WBCs(x10^3^/mm^3^)	8.99 ± 0.38	6.55 ± 0.41	0.007*
CEA (ng/ml)	36.55 ± 9.02	1.85 ± 0.27	0.09
CA 19-9 (U/ml)	271.05 ± 166.06	5.25 ± 1.16	0.48
miR-576 delta Ct	3.97± 0.21	2.38 ± 0.24	0.002*
miR-613 delta Ct	4.15 ± 0.21	2.69 ± 0.30	0.003*
NDRG2 (pg/ml)	3.03 ± 0.18	3.17 ± 0.54	0.75
YKL40 (pg/ml)	3813.40 ± 251.38	731.50 ± 119.35	<0.001*

Data were expressed as mean ± standard error of mean; *, is significant.

**Table 4:** Data Analysis of miR-576-3p in the Studied Groups

Group (%)	GAPDH Ct	miR-576-3p Ct	∆Ct	∆∆Ct	Fc
Control	19.45	21.83	2.38	0	1
Total patients	20.86	24.83	3.97	1.59	0.33
Grade:					
II (86.96%)	20.93	24.91	3.98	1.6	0.33
III (13.04%)	20.23	24.09	3.86	1.48	0.36
Stage:					
I (13.04%)	19.25	24.81	5.56	3.18	0.11
IIA (30.43%)	21.64	27.18	5.54	3.16	0.11
IIIA(13.04%)	19.85	24.38	4.53	2.15	0.23
IVA (13.04%)	19.94	23.83	3.89	1.51	0.35
IIB (4.35%)	20.93	24.86	3.93	1.55	0.34
IIIB (21.75%)	21.27	25.83	4.56	2.18	0.22
IIIC (4.35%)	21.76	25.24	3.48	1.10	0.47
Tumor Site:					
Colon (65.22%)	20.76	24.69	3.93	1.55	0.34
Rectum (30.43%)	21.06	25.2	4.14	1.76	0.3
Colon & Rectum (4.35%)	21.76	25.24	3.48	1.10	0.47
Lymph Node:					
Positive (56.52%)	20.78	25.22	4.44	2.06	0.24
Negative (43.48%)	20.84	24.8	3.96	1.58	0.33

GAPDH, Glyceraldehyde-3-phosphate dehydrogenase; Ct, Cycle threshold; Fc, Fold change.

**Table 5:** Data Analysis of miR-613 in the Studied Groups

Group (%)	GAPDH Ct	miR-613 Ct	∆Ct	∆∆Ct	Fc
Control	19.45	22.14	2.69	0	1
Total patients	20.86	25.01	4.15	1.46	0.36
Grade:					
II (86.96)	20.93	25.11	4.18	1.49	0.36
III(13.04%)	20.23	24.13	3.9	1.21	0.43
Stage:					
I (13.04%)	19.25	24.32	5.07	2.38	0.19
IIA (30.43%)	21.64	25.44	3.8	1.11	0.46
IIIA (13.04%)	19.85	24.11	4.26	1.57	0.34
IVA (13.04%)	19.94	24.47	4.53	1.84	0.28
IIB (4.35%)	21.94	25.16	3.22	0.53	0.69
IIIB (21.75%)	21.27	26.31	5.04	2.35	0.2
IIIC (4.35%)	21.76	24.72	2.96	0.27	0.83
Tumor Site:					
Colon (65.22%)	20.76	24.87	4.11	1.42	0.37
Rectum (30.43%)	21.06	25.49	4.43	1.74	0.3
Colon & Rectum (4.35%)	21.76	24.72	2.96	0.27	0.83
Lymph Node:					
Positive (56.52%)	20.78	25.26	4.48	1.79	0.29
Negative (43.48%)	20.84	24.96	4.12	1.43	0.37

GAPDH, Glyceraldehyde-3-phosphate dehydrogenase; Ct, Cycle threshold; Fc, Fold change

**Table 6:** Serum Mean Levels of Parameters According to the Clinicopathological Data of the Patients

Grade	miR-576-3p	miR-613	NDRG2	YKL40
II	0.54 ± 0.06	0.54 ± 0.05	2.88 ± 0.18	3742.56 ± 264.18
III	0.50 ± 0.19	0.50 ± 0.14	4.34 ± 0.76	4923,33 ± 26,67
*P*-Value	0.82	0.83	0.02*	0.27
Stage				
I	0.14 ± 0.05	0.41 ± 0.19	2.97 ± 0.30	5125.00 ± 72.17
IIA	0.67 ± 0.12	0.64 ± 0.10	3.26 ± 0.21	3220.00 ± 516.68
IIIA	0.23 ± 0.02	0.35 ± 0.05	4.33 ± 0.76	3502.00 ± 870.51
IVA	0.56 ± 0.19	0.50 ± 0.17	2.28 ± 0.23	5116,67 ± 158.33
IIB	0.69 ± 0.03	0.69 ± 0.03	2.36 ± 0.06	3525.00 ± 75.00
IIIB	0.43 ± 0.14	0.35 ± 0.11	3.10 ± 0.48	3312.00 ± 659.80
IIIC	0.47 ± 0.02	0.83 ± 0.03	1.65 ± 0.05	2370.05 ± 0.05
*P*-Value	0.09	0.3	0.04*	0.28
Tumor Site				
Colon	0.56 ± 0.06	0.56 ± 0.06	2.89 ± 0.19	3727.43 ± 300.50
Rectum	0.49 ± 0.12	0.43 ± 0.08	3.63 ± 0.49	4266.92 ± 508.02
Colon & rectum	0.47 ± 0.02	0.83 ± 0.03	1.65 ± 0.05	2370.05 ± 0.05
P-Value	0.85	0.24	0.33	0.09
Lymph Node				
Positive	0.39 ± 0.07	0.43 ± 0.06	3.12 ± 0.30	3732.73 ± 399.47
Negative	0.57 ± 0.10	0.59 ± 0.09	3.05 ± 0.18	3453.57 ± 376.05
*P*-Value	0.12	0.14	0.86	0.64

Data were expressed as mean ± standard error of mean; *, is significant.

**Table 7:** Correlation between Parameters in the CRC Patients

	CEA	CA19-9	miR-576-p	miR-613	NDRG2
CA19-9					
*r*	-0.04				
*p*	0.75				
miR-576-3p					
*r*	-0.3	-0.21			
*p*	0.82	0.1			
miR-613					
*r*	-0.07	-0.27	0.75		
*p*	0.56	0.04*	<0.001**		
NDRG2					
*r*	-0.37	-0.01	-0.12	-0.16	
*p*	0.004**	0.96	0.35	0.23	
YKL40					
*r*	-0.21	0.17	0.15	0.13	0.1
*p*	0.13	0.23	0.3	0.34	0.44

*, is significant; **, is highly significant; r, Pearson’s correlation coefficient; p, probability value.

**Table 8:** The Sensitivity, Specificity, Cut off Value, and AUC (area under curve) for NDRG2 and YKL40 in Colorectal Cancer Patients

Parameters	Sensitivity (%)	Specificity (%)	Cut off Value	Area Under Curve	*P*-Value
NDRG2	60	66.7	<2.5	0.54	0.64
YKL40	96	91.7	<1200	0.97	0.0001

## Discussion

Colorectal cancer (CRC) still a significant medical issue in the world (Bray et al., 2018). CRC rate in Egypt is expected to be elevated by 2025 (Ou et al., 2011). CRC diagnosis depends mainly on colonoscopy which is uncomfortable and invasive. The screening procedure involves different markers, for example, the serum CA19-9 and CEA, fecal occult blood testing (FOB); however, these markers have low sensitivity and specificity (Burch et al., 2007). Thus, new non-invasive markers with high sensitivity and specificity are critically required for the CRC diagnosis. For example, micro RNAs that have been recorded to have a significant role in oncogenesis (Chen et al., 2008). Our study was done on miR-576-3p, miR-613and NDRG2 protein which have an apoptotic property in cancerous tissues and YKL40 which regarded as an antiapoptotic marker.

miR-576-3p which is considered as a newly discovered miRNA is downregulated in bladder cancer (Liang et al., 2015). The miR-576-3p higher expression could down-regulate the *CCND1* gene expression, also it could inhibit the colony formation, cell viability, in addition promote G1-phase arrest in UM-UC-3 and T24 cells. The down-regulation of miR-576-3p raised the CCND1 gene expression and induced the bladder cancer cells multiplication by accelerating the cell cycle progression. So miR-576-3p played a role as a tumor suppressor in bladder cancer cells by suppressing the *CCND1* gene expression.* miR-576-3p* overexpression in cells with bladder cancer can promote G1-phase arrest (Liang et al., 2015). This is agreed with our study results as the *miR-576-3p* expression was down-regulated in patients with colorectal malignancy by fold change (Fc= 0.33).

miR-613 could inhibit cancer cell proliferation, migration, invasion and promotes apoptosis by targeting numerous oncogenes, including DCLK1 (Wang et al., 2016), Fzd7 (Ren et al., 2016), and SphK2 (Qiu et al., 2016).miR-613 had been stated to be down-regulated in different cancers and act as a tumor suppressor (Wang et al., 2016). These cancers include lung cancer, gastric cancer, esophageal squamous cell cancer (Guan et al., 2016), prostatic cancer, and ovarian malignancy(Fu et al., 2016). In ovarian cancer, miR-613 suppressed cell proliferation, invasion and colony formation by targeting *KRAS* expression (Fu et al., 2016). In prostate cancer, miR-613 inhibited prostate cancer invasion and cell proliferation by down-regulating Wnt signaling pathway and suppressing Frizzled7 (Ren et al., 2016). In non-small lung cancer, miR-613 inhibited cell cycle, proliferation and colony formation by targeting CDK4 inhibition (Li et al., 2016). With agreement with these findings, our study results showed that *miR-613 *expression was down-regulated in CRC patients as compared to control by fold change (0.363).

*NDRG2* which is a member of *NDRG* family is reported to act as a tumor suppressor gene (Oh et al., 2012). It has been reported that levels of *NDRG2 *expression are low in breast cancer cells compared to that in normal tissues (Ma et al., 2012). NDRG2 overexpression revokes the genes up-regulation related with the G protein signaling pathway and the down-regulation of sets of genes identified with the cell cycle at M phase that is appropriate with cell cycle investigations (Liu et al., 2012). A signaling pathway investigation illustrated diminished biosynthesis of glycosyl phosphatidyl inositol (GPI) - anchor and degradation of protein (Liu et al., 2012). It’s declared that the expression of NDRG2 can promote G1 arrest (Ma et al., 2010). Also, the arrest of cell cycle at G1/S was occurred after NDRG2 was introduced into SW620 cells (Kim et al., 2009). So NDRG2 can inhibit the cell cycle resetting in tumors. In our study, it was observed that the levels of NDRG2 were decreased in patients with CRC compared to control (3.03, 3.17; respectively) but the decrease wasn’t reach to the significance level. This is with agreement with this reference (Feng et al.,2011) which reported that, NDRG2 mRNA expression level is decreased in CRC compared to those in normal colonic tissues in the control group. 

YKL-40 is a conserved glycoprotein synthesized by tumor cells (including CRC cells), neutrophils and macrophages (Johansen et al., 2009) and embryonic tissues (Johansen et al., 2007).YKL40 is considered to have a significant role in the differentiation and proliferation of tumor cells support cell survival by stimulating protein kinase B (AKT)and suppress apoptosis (Chen et al., 2011), activate angiogenesis (Faibish et al., 2011), influence extracellular tissue renovation (Johansen et al., 2006), and considered as one of the most significant growth factors for fibroblasts (Recklies et al., 2002). YKL40 is secreted by inflammatory cells and several solid tumors involving colon, breast, lung, glioblastoma, ovary, prostate, and kidney tumors (Culig et al., 2004).. In prostate cancer, elevated serum levels of YKL40 had been reported in primary prostate cancer patients when compared with benign prostate hyperplasia patients, considering that YKL40 may affect the aggressiveness and progression of prostate cancer (Kucur et al., 2008). In our study, the serum YKL40 level in colorectal cancer patients was significantly increased compared to control (p-value < 0.001).

In conclusion, our study concluded that the assessment of miR-576-3p, miR-613, and YKL40 levels may have a great value in the diagnosis of CRC and another study with large number of subjects is needed to estimate the accurate prognostic significance of these biomarkers.
